# Mechanistic insights into clary sage oils role in wound healing: targeting oxidative stress and inflammatory pathways

**DOI:** 10.3389/fphar.2025.1567205

**Published:** 2025-08-15

**Authors:** Rehab Ahmed, Krishnaraju Venkatesan, Durgaramani Sivadasan, Nizar Sirag, Hassabelrasoul Elfadil, Gayathri Anbumani, Debadrita Saha

**Affiliations:** ^1^ Division of Microbiology, Immunology and Biotechnology, Department of Natural Products and Alternative Medicine, Faculty of Pharmacy, University of Tabuk, Tabuk, Saudi Arabia; ^2^ Department of Pharmacology, College of Pharmacy, King Khalid University, Abha, Asir, Saudi Arabia; ^3^ Department of Pharmaceutics, College of Pharmacy, Jazan University, Jazan, Saudi Arabia; ^4^ Department of Natural Products and Alternative Medicine, Faculty of Pharmacy, University of Tabuk, Tabuk, Saudi Arabia; ^5^ Kaarunee VRK Life Sciences, Chennai, India; ^6^ Department of Pharmaceutical Sciences, SRM College of Pharmacy, Kattankulathur, Tamil Nadu, India

**Keywords:** clary sage oil, *Salvia sclarea*, wound healing, inflammatory cytokines, homeostasis

## Abstract

**Introduction:**

Clary sage essential oil (CSEO), extracted from Salvia sclarea, is known for its antioxidant and anti-inflammatory properties. While its general therapeutic benefits are recognized, its specific role in wound healing remains inadequately explored. This study investigates the effects of CSEO on oxidative stress, inflammatory cytokines, and tissue regeneration in a rat excision wound model.

**Methods:**

Rats were subjected to full-thickness excision wounds and divided into treatment, reference and control groups. The treatment group received topical application of CSEO, while reference and controls received standard drug and soft paraffin respectively. Wound healing was evaluated by measuring wound contraction and epithelialization. Biochemical assays assessed oxidative stress and inflammatory markers, while immunohistochemistry was used to analyse CD68 expression. Histological examinations evaluated granulation tissue, collagen deposition, angiogenesis, and inflammatory infiltration.

**Results:**

CSEO significantly enhanced wound contraction and epithelialization compared to controls. Biochemical analysis showed a reduction in oxidative stress markers. Immunohistochemical evaluation revealed decreased CD68 expression, along with significantly lower levels of pro-inflammatory cytokines TNF-α and IL-6. Histopathological findings demonstrated improved granulation tissue formation, increased collagen synthesis, enhanced angiogenesis, and reduced inflammatory cell presence in CSEO-treated wounds.

**Discussion:**

CSEO facilitates wound healing through its antioxidant and anti-inflammatory actions. The reduction in oxidative stress and inflammatory mediators, along with improved histological parameters, suggests that CSEO supports effective tissue repair. These findings highlight the therapeutic potential of CSEO as a natural wound-healing agent and support its further investigation in regenerative medicine.

## 1 Introduction

The wound healing process is intricate and dynamic and progresses through distinct but overlapping stages: hemostasis, inflammation, proliferation, and maturation. Effective wound healing is crucial for restoring skin integrity and preventing complications such as infections and chronic wounds (Kostova et al., 2020). Despite advancements in medical treatments and wound care strategies, exploring both traditional and alternative therapies, particularly plant-based ones, remains an active area of research. Among these, essential oils have garnered attention due to their diverse bioactive properties and potential roles in promoting tissue repair.

One such essential oil is Clary sage essential oil (CSEO), derived from *Salvia sclarea*, a plant renowned for its therapeutic versatility and potential to enhance wound healing (El-Gohary et al., 2020). CSEO is extracted from the flowers and leaves of *S. sclarea*, the Lamiaceae family native to the Mediterranean region (Zhumaliyeva et al., 2023). It has a distinctive aroma and is rich in bioactive constituents such as linalool, linalyl acetate, geraniol, and other terpenes, demonstrating anti-inflammatory, antibacterial, and analgesic effects (Agnihotry et al., 2024). These multifaceted pharmacological effects make CSEO an attractive candidate for wound care, as it may accelerate healing, reduce infection risk, and alleviate pain (Kolimi et al., 2022).

Current wound management strategies frequently rely on synthetic agents, including antibiotics and corticosteroids. However, their prolonged use may lead to delayed healing, microbial resistance, and systemic side effects. These limitations underscore the need for safer, more holistic alternatives (Falih and Al-Ali, 2024). Plant-derived therapies, particularly essential oils, have emerged as promising options due to their multi-targeted mechanisms, combining antimicrobial, anti-inflammatory, and regenerative actions. CSEO, therefore, represents a novel and natural alternative with the potential to overcome the drawbacks of conventional therapies (Falih and Al-Ali, 2024; Raveau et al., 2021).

Multiple intrinsic and extrinsic factors influence wound healing, such as infection, inflammation, and impaired blood flow. Evidence suggests that CSEO can modulate many of these factors (Falih and Al-Ali, 2024). Several studies have reported its ability to promote skin regeneration, enhance circulation, and reduce microbial colonization at the wound site. This makes it a viable complement or alternative to traditional wound treatments often dominated by synthetic agents (Raveau et al., 2021).

The antimicrobial effects of CSEO, primarily attributed to its constituents like linalool and linalyl acetate, are especially valuable in preventing wound infections, a major complication that can delay healing and worsen outcomes (Sienkiewicz et al., 2015). Research has demonstrated its antibacterial activity against Gram-positive and Gram-negative bacteria, supporting its use in infection control for open wounds (Orchard and van Vuuren, 2017). Additionally, the anti-inflammatory properties of CSEO help modulate the inflammatory phase of wound healing by reducing excessive edema and promoting a more balanced healing response (Trinh et al., 2022).

CSEO regenerative capabilities are equally noteworthy. Studies indicate that it can stimulate the production of collagen and elastin proteins essential for tissue repair and remodelling (Mahboubi, 2020). The topical application of CSEO has been shown to accelerate wound closure and enhance new tissue formation in animal models. Moreover, promoting vasodilation improves blood flow to the wound site, facilitating the delivery of oxygen and nutrients essential for repair (Mori et al., 2016). The analgesic effects of CSEO further enhance its therapeutic value. By alleviating pain during the inflammatory phase, it improves patient comfort. These soothing effects also support its traditional use in aromatherapy for stress and anxiety relief, suggesting added benefits to overall wellbeing during recovery (Mitic et al., 2020).

Although the anti-inflammatory and antioxidant effects of CSEO have been previously reported, comprehensive evaluations of its molecular and histological impacts on wound healing are limited. Specifically, few studies have investigated its influence on cytokine expression, oxidative stress markers, and histopathological restoration. Moreover, most existing studies have focused primarily on macroscopic wound closure, with limited insight into underlying biochemical and tissue-level changes. Additionally, variations in formulation and dosing leave important gaps regarding standardization and reproducibility. This body of data must be strengthened to confirm the therapeutic significance of CSEO and get a more thorough knowledge of the mechanisms of action.

Despite positive findings, it is crucial to remember that most CSEO research wound-healing effects have been conducted in animal models (Son et al., 2025). Consequently, although initial results are encouraging, more studies are necessary to ascertain the ideal dose, administration techniques, and safety profiles for human use (Orchard et al., 2018). This study examines the wound-healing efficacy of CSEO using an animal model, with a specific focus on evaluating wound closure percentage, TNF-α, IL-1β, histopathological changes, oxidative stress markers such as ROS, MDA, GSH, SOD, and re-epithelialization.

## 2 Materials and procedures

### 2.1 Experimental animals

Male albino rats weighing 150–170 g and between the ages of 8 and 12 weeks were obtained from the Central Animal House. Rats were acclimated to a humidity level of 55% and a 25°C ± 2°C temperature. The ongoing evaluation of rats was performed utilizing 12-hour cycles of illumination and obscurity. Rats were accommodated in distinct enclosures and given a standard laboratory meal and unrestricted access to tap water. All animal methods adhered to the rules established by the Institutional Animal Ethics Committee (IAEC) of King Khalid University, which authorized all experimental protocols (ECM/2021-5306) (Sujatha et al., 2024).

### 2.2 Oil source

The essential oil is obtained from BMV Fragrances Pvt Ltd, Uttar Pradesh. The certificate of analysis indicates that the oil is obtained via steam distillation. The primary constituents of the oil are Linalool, Linalyl Acetate, and Germacrene D.

### 2.3 Identification of chemical substances in CSEO using GC-MS

The chemical composition of CSEO was analyzed utilising a Shimadzu GCMS-QP2010 Plus system (Shimadzu Corporation, Kyoto, Japan) in conjunction with an HP-5MS capillary column (30 m, 0.25 mm i.d., 0.25 m film thickness, Agilent Technologies, Santa Clara, CA, USA) by gas chromatography-mass spectrometry (GC-MS). Before analysis, the material was filtrated via a 0.22 µm membrane filter following dilution in hexane (1:10 v/v). At a temperature of 250°C, a 40:1 split ratio was used to inject a 1.0 µL aliquot in split mode. Helium operated as the carrier gas at a constant 1.0 mL/min flow rate. Starting at 50°C (held for one minute), the oven temperature increased by 8°C each minute to 280°C, which it maintained for two minutes. At 200°C, the ion source temperature was set, and electron impact (EI) at 70 eV was used to ionise the material. Over 31 min, mass spectra were acquired in the 40–900 m/z region. A comparison was made between the mass spectra of chemical compounds and the NIST Library Version 8.0 (National Institute of Standards and Technology, Gaithersburg, Maryland, United States of America) to determine the identities of the compounds.

### 2.4 Ointment preparation

Ointments were formulated by integrating 10% w/w of CSEO into soft paraffin sourced from Zuche Pharmaceuticals, New Delhi. Ligation was employed to achieve a smooth and uniform texture by formulating the ointment on a slab. Soft paraffin was the negative control; silver sulfadiazine ointment (1% w/w) was the standard.

### 2.5 Toxicity studies

Ten male Sprague Dawley rats weighing 250–300 g were utilised for skin irritation evaluation by OECD guideline 404 (OECD, 1981). The animals were randomly allocated into two groups of five rats each: a test group (n = 5) with a 10% essential oil formulation and a negative control group (n = 5) receiving sterile water. The hair from the rear area of the rats was removed using a sterile razor to a diameter of approximately 20 cm at the lower mid-position, after which the rats were separately housed. The rats were kept undisturbed for 24 h. A 10% essential oil solution was consistently applied to the depilated region of the test individuals. The plant extracts were administered to the skin using gauze and a non-irritating adhesive tape for one hour, following which they were removed, and the skin surface was washed with distilled water and evaluated for irritation. Observations were conducted at 24, 48, and 72 h following application, in line with the OECD procedure. Similarly, sterile cotton soaked in a sufficient amount of sterile water was used to apply sterile water topically to the control rats, who were then wrapped in gauze and non-irritating tape. We used the Draize grading technique to evaluate erythema and oedema. Little erythema or oedema is indicated by a score of 1, whilst the absence of either condition is indicated by a score of 0. A score of 2 signifies substantial oedema, marked by elevated skin at the edges of the impacted area. Serious erythema or oedema is indicated by a score of 4, whereas moderate to severe erythema or oedema is indicated by a score of 3 (Draize, 1959).

### 2.6 Excision wound model

#### 2.6.1 Experimental design

Each group included 6 rats (*n* = 6), and the excision wound model experiment was conducted once with six biological replicates per group. All animals were housed individually to avoid wound interference.

Group 1 — Control group (treated with soft paraffin).

Group 2 — Standard treatment (1% w/w silver sulfadiazine) was given to the animals.

Group 3 — Animals administered the test substance (10% w/w CSEO).

#### 2.6.2 Wounding (Morton and Malone method)

The excision wound model was developed using three cohorts of albino rats. To create anaesthesia, animals received intraperitoneal injections of xylazine (10 mg/kg) and ketamine (50 mg/kg) after a 12-hour fast. Rats were placed in the prone position on a sterile surgical platform, and the dorsal region between the two scapulae was shaved using an electric clipper. The area was disinfected with 70% ethanol to maintain aseptic conditions.

A circular full-thickness excision incision measuring roughly 2 cm in diameter (area: ∼200 mm^2^) and 2 mm in depth was made on the dorsum using a sterile surgical blade, by the technique established by Morton and Malone (1972). This model was chosen because it reliably mimics human cutaneous wound healing by involving all skin layers (epidermis, dermis, and part of the hypodermis) and allows for consistent re-epithelialization and wound contraction measurement. Excess blood was absorbed using sterile gauze. All wounds were left unbandaged and exposed to the air to simulate natural healing conditions. Animals were monitored daily, and any signs of infection or illness were removed from the study. Each rat was housed in a cage to prevent interference with the wound site (Murugesu et al., 2021).

The test formulation CSEO at 10% w/w in soft paraffin, was applied topically once daily until complete wound closure. In practice, essential oils are often diluted to concentrations between 1% and 10% for topical use (Elshamy et al., 2020), depending on the desired therapeutic effect and the potential for skin irritation. A 10% w/w concentration of CSEO falls within this commonly accepted range, balancing efficacy with safety. The once-daily application schedule was chosen to maintain a consistent therapeutic effect while minimizing stress and handling-related variables.

### 2.7 Treatment

Using a sterile spatula, the test and regular medication were administered every day until the epithelium was completely covered. Each day, this was executed with heightened vigilance to avert dose discrepancies. The surfaces of each wound were meticulously cleansed with a tampon saturated in physiological serum before the administration of treatments. Treatments were applied once daily to each animal (n = 6 per group). The excision wound model experiment was conducted with six biological replicates per group. Wound areas were measured independently for each rat at specified time points (days 1, 4, 8, 12, 16, and 21), and each measurement was recorded in duplicate to ensure accuracy and reproducibility. Graph paper was utilized to quantify the documented wound areas on days 1, 4, 8, 12, 16, and 21. Epithelization is where the eschar detaches, leaving no exposed wound. Upon completion of the research, the animals were euthanized. On day 21, the healed wound and adjacent skin were excised and split into two equal portions for histopathological analysis and the antioxidants measurement procedure. Treatments were administered daily to each animal (*n* = 6 per group). The wound area for each rat was measured independently at specified time points. All measurements were taken in duplicate to ensure accuracy.

### 2.8 Evaluation parameters

Wound contraction percentage: The measurement of wound contraction was used for assessment. Every four days (days 1, 4, 8, 12, 16, and 21) the wound was marked with a permanent marker and clear paper to gauge the rate of closure. The area (mm^2^) of each trace inside its borders was estimated using planimetry. All wounds were digitally documented at consistent intervals. Measuring the wound area was conducted using Adobe Photoshop and ImageJ tools (Murugesu et al., 2021).

% wound contraction= ((wound area on day zero - wound area on specific day)/wound area on day zero) × 100 (Murugesu et al., 2021).

### 2.9 Biochemical evaluation

#### 2.9.1 Sample collection

Blood samples were obtained from all animals in each group (n = 6 per group) using the orbital sinus method in EDTA-free tubes. Sera were separated and stored to quantify CD68, TNF-α, tumour necrosis factor-alpha, and interleukin-1 beta (IL-1β). Each serum sample represented a biological replicate. All biochemical assays were performed in technical duplicates to ensure the accuracy and reproducibility of the data (Al-Salih and Al-Jameel, 2023).

#### 2.9.2 Assessment of TNF-α and IL-1β concentrations in rat serum

Serum levels of TNF-α and IL-1β were quantified using commercial ELISA kits from Elabscience (Cat. No: E-EL-H0109 for TNF-α and E-CL-R0012 for IL-1β), following the manufacturer instructions. A standard curve was used to calculate the concentrations of cytokines and reported in pg/mL. Samples from each animal (n = 6 per group) were analyzed individually, with all assays performed in duplicate to ensure accuracy (Murugesu et al., 2021; Pereira Beserra et al., 2020).

#### 2.9.3 Estimation of CD68 in the rat serum

A commercial ELISA kit from MyBioSource (Cat. No: MBS705029) was employed to quantify CD68 levels in the serum of each animal (n = 6 per group), according to the guidelines provided by the manufacturer. The outcomes were quantified using a standard curve and expressed in ng/mL. Each assay was conducted in duplicate for each sample (Al-Salih and Al-Jameel, 2023).

#### 2.9.4 Antioxidant activity

Skin tissue specimens from all rats (n = 6 per group) were homogenized in 1 mL saline per Gram of tissue using a glass homogenizer. Before further analysis, The homogenates were centrifuged for 30 min at 4°C at 10,000 g. The supernatants that were produced were then stored at 80°C. Levels of reactive oxygen species (ROS), glutathione (GSH), malondialdehyde (MDA), and superoxide dismutase (SOD) were evaluated using particular ELISA kits, following the respective manufacturers’ instructions: ROS (BT Lab, USA), MDA (Eagle Biosciences, Cat. No: LIP39-K01), GSH (Eagle Biosciences, Cat. No: GLU39-K01), and SOD (Cusabio, Cat. No: CSB-EL022397RA). Each sample was analyzed in duplicate (Murugesu et al., 2021; Pereira Beserra et al., 2020).

### 2.10 Histopathological evaluation

#### 2.10.1 Sample collection

We prepared and examined tissue samples from every animal (n = 6 per group). Every animal-removed tissue sample was preserved in buffered formalin and then put through a series of xylene and alcohol gradations before being embedded in paraffin blocks. Collagen fibre density was determined by regularly staining 4 µm thick tissue slices using a specific Masson’s trichrome and hematoxylin/eosin. Leica Microsystems, Wetzlar, a light microscope, and the Leica Application Suite were used to view and take pictures of mounted slides. Representative images from all species were analyzed. To guarantee uniformity, histological characteristics were evaluated in duplicate sections from every sample.

### 2.11 Statistical analysis

The findings were presented as means ± SEM. The data were analysed using GraphPad Prism software (version 8.00) and subjected to Tukey’s comparison test following one-way analysis of variance (ANOVA). The significance level was set at p < 0.05.

## 3 Results

The results indicated a significant difference in wound healing among the groups. Wounds in the reference and control groups necessitated up to 20 days for healing, whereas those treated with CSEO exhibited a notably accelerated recovery, achieving full closure within just 18 days. This highlights the potent therapeutic efficacy of CSEO in promoting faster wound healing.

### 3.1 Essential oil chemical constituents

**FIGURE 1:**
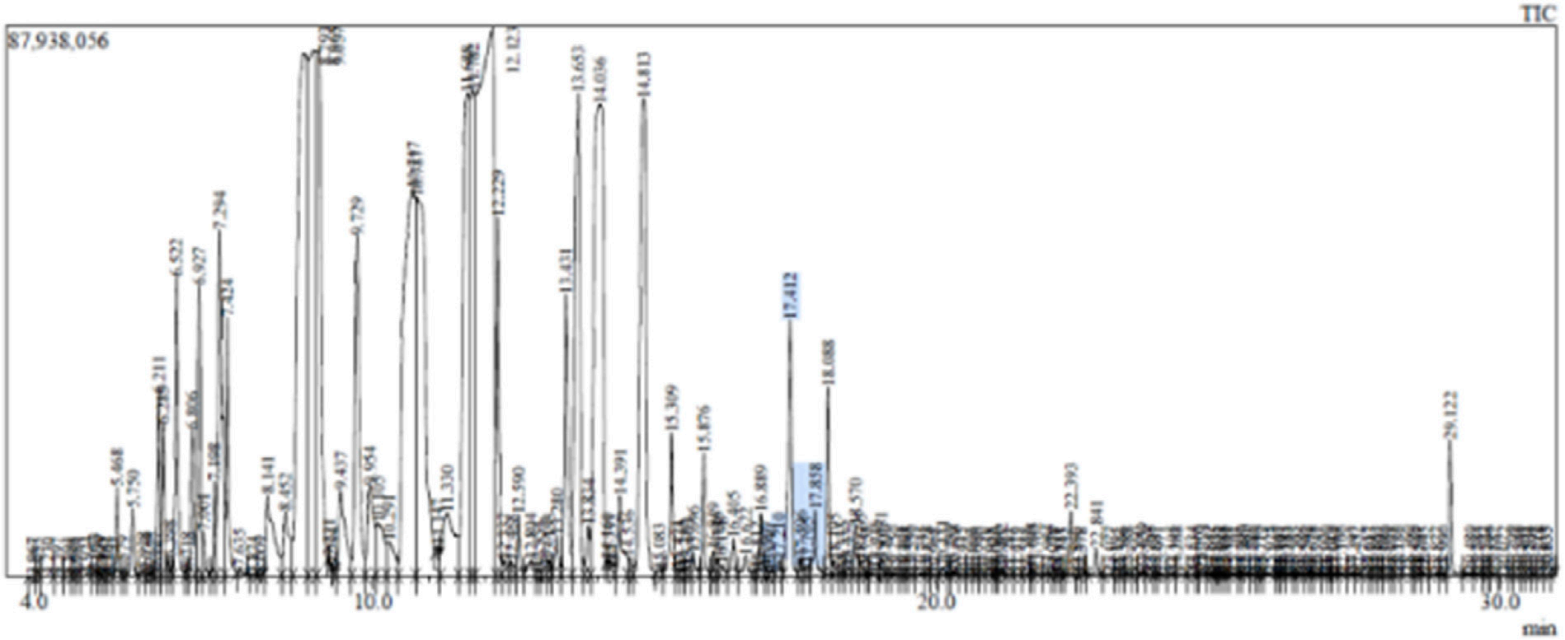
GCMS data of CSEO.

**FIGURE 2:**
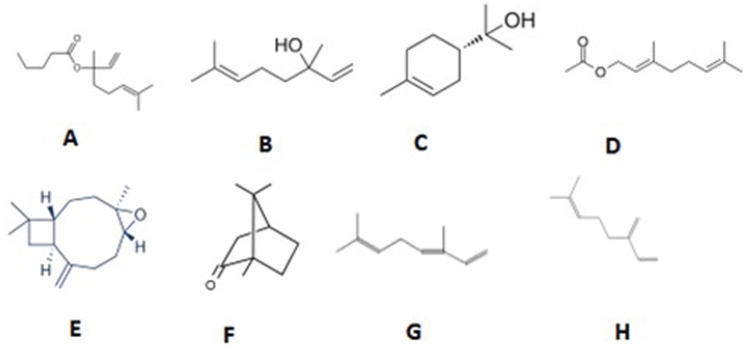
Structure of major compounds identified in an CSEO. **(A)** Linalyl isobutyrate, **(B)** Linalool, **(C)** Alpha-terpineol, **(D)** Geranyl acetate, **(E)** Caryophyllene oxide, **(F)** Camphor, **(G)** Beta-ocimene, **(H)** Beta-Myrcene.

Analysis of the GC-MS data of the (Table 1; Figures 1, 2) of MEO identified a diverse array of volatile constituents, predominantly comprising oxygenated monoterpenes and esters. The major components detected were linalyl isobutyrate (18.88%), linalool (15.86%), alpha-terpineol (13.01%), and geranyl acetate (10.87%), indicating a monoterpene-rich profile. Other notable constituents included caryophyllene (6.72%), camphor (2.37%), beta-ocimene (2.48%), and beta-myrcene (1.39%), along with minor amounts of terpinyl acetate, dihydro-linalool, and caryophyllene oxide. The overall composition reflects a complex mixture dominated by alcohols, esters, and sesquiterpenes, with a strong representation of compounds known for their aromatic and bioactive properties. These constituents’ presence and relative abundance provide insight into the phytochemical signature of the analyzed oil and its potential therapeutic applications.

**TABLE 1:** Compounds exhibiting a peak area percentage >1.0% from the CSEO GCMS data.

Retention duration (min)	Compound designation	Peak area (%)	Molecular formula	Chemical class
6.522	7-Methyl-3-Methylene-1,6-Octadiene	1.39	C_10_H_16_	Beta-Myrcene/Monoterpenoids
6.927	1,3,6-Octatriene, 3,7-Dimethyl-, (E)-	1.26	C_10_H_16_	beta-ocimene/Monoterpenes
7.294	Cyclohexene, 1-Methyl-4-(1-Methylethenyl)-	2.09	C_10_H_16_	Limonene/Monoterpenes
7.424	Beta-Ocimene	1.22	C_10_H_16_	beta-ocimene
8.793	1,6-Octadien-3-Ol, 3,7-Dimethyl-	8.50	C_10_H_18_O	Linalool/Monoterpenes
8.965	1,2,4,5,9,10-Triepoxydecane	6.19	C_10_H_16_O3	Triepoxy decane
9.037	3,7-Dimethylocta-1,6-dien-3-ol	3.74	C_10_H_18_O	Linalool/Monoterpenes
9.729	Camphor	2.37	C_10_H_16_O	Camphor/Ketones
10.717	3-Cyclohexene-1-Methanol.,.Alpha.,.Alpha.,4-Trimethyl-	7.09	C_10_H_18_O	Alpha-Terpineol/Monoterpenes
10.787	lpha.-Terpineol	5.92	C_10_H_18_O	alpha-Terpineol/Monoterpenes
11.330	11-Dodecyn-1-ol acetate	1.19	C_14_H_26_O_2_	11-Dodecyn-1-ol acetate
11.688	3,7-Dimethyl-1,6-octadien-3-yl isobutyrate	5.06	C_14_H_24_O_2_	Linalyl isobutyrate
11.782	1,6-Octadien-3-Ol, 3,7-Dimethyl-	3.62	C_10_H_18_O	Linalool/Monoterpenes
12.123	3,7-Dimethyl-1,6-octadien-3-yl isobutyrate	13.82	C_14_H_24_O_2_	Linalyl isobutyrate
12.229	6-Octen-3-Ol,3,7-Dimethyl-	1.49	C_10_H_20_O	Dihydrolinalool
13.431	alpha-Terpinyl acetate	1.66	C_12_H_20_O_2_	Terpinyl acetate/Terpenes
13.653	2,6-Octadien-1-ol, 3,7-dimethyl-, acetate	3.79	C_12_H_20_O_2_	Geranyl Acetate/Acetates
14.036	2,6-Octadien-1-Ol, 3,7-Dimethyl-, Acetate	7.08	C_12_H_20_O_2_	Geranyl Acetate
14.813	Bicyclo[7.2.0]Undec-4-Ene, 4,11,11-Trimethyl-8-Methylene-,	5.22	C_15_H_24_	Caryophyllene /Polycyclic Sesquiterpenes
17.412	-)-5-Oxatricyclo [8.4.0.0(4,6)]Dodecane,,12-Trimethyl -9-Meth	1.50	C_15_H_24_O	Caryophyllene oxide

### 3.2 Toxicity studies

The test oil was safe at a dosage of 10% w/w in the acute cutaneous irritation research; when the animals were monitored for 3 days following the experiment, there were no indications of skin response, inflammation, erythema, irritation, or redness, nor any other negative reaction.

### 3.3 Percentage wound contraction

**FIGURE 3:**
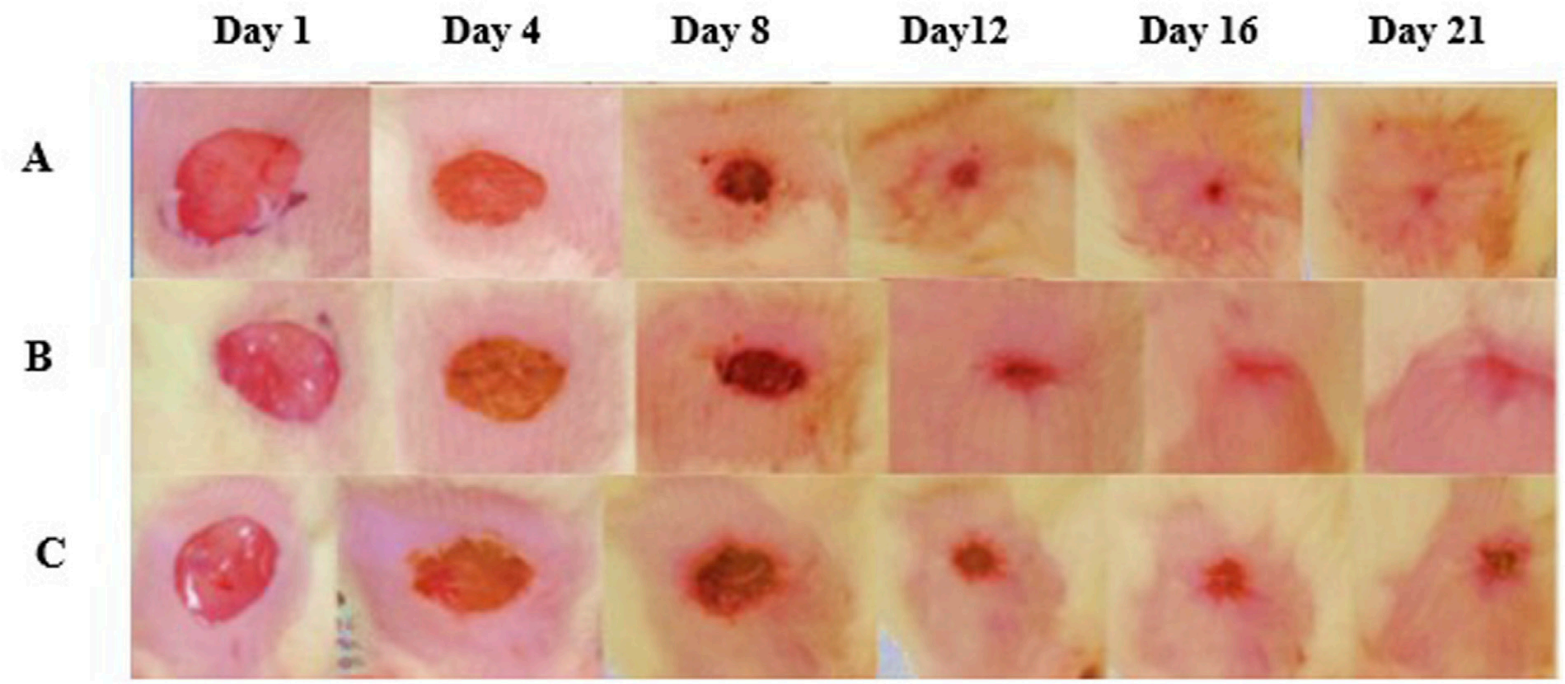
Wound healing images among many groups. **(A)** The treatment group, **(B)** reference, and **(C)** control demonstrated effective wound healing, as seen by representative photos taken from 1st day to the 21st day. The therapy group exhibited expedited wound healing, attaining epithelialization completely at the conclusion of the observation period.

The wound closure % was assessed on days 4, 8, 12, 16, and 20 to evaluate the wound healing efficacy of CSEO, as compared to the reference and control groups (Table 2; Figure 3). On day 4, the CSEO-treated group exhibited significantly enhanced wound contraction (35.90% ± 3.334%) against the control (*p < 0.05), and this trend continued throughout the observation period. By day 12, CSEO treatment resulted in 92.47% ± 0.8033% (***^#^ *p* < 0.005) closure, which was markedly higher than both the control (84.45% ± 0.7978%) and reference groups (87.06% ± 1.798%, ^#^ *p* < 0.005). Wound healing in the CSEO group was nearly complete by day 20 (99.89% ± 0.1054%, ***^#^ *p* < 0.005), significantly surpassing the reference (99.03% ± 0.1849%, *^#^ *p* < 0.05) and control groups (98.50% ± 0.2844%, ***^#^ *p* < 0.005). The reference group also demonstrated moderate improvement over the control (*^#^ *p* < 0.05 on days 12–20). These findings establish the superior wound-healing potential of CSEO.

**TABLE 2:** Impact of CSEO on wound closure percentage against reference and control groups.

Groups	Wound closure percentage (Days)				
	4th day	8th day	12th day	16th day	20th day
CSEO	35.90 ± 3.334**	54.57 ± 1.302*	92.47 ± 0.8033***^#^	98.80 ± 0.2505***^#^	99.89 ± 0.1054***^#^
Reference	14.55 ± 2.624***	45.16 ± 3.109*^#^	87.06 ± 1.798*^#^	96.23 ± 0.5274*^#^	99.03 ± 0.1849*^#^
Control	21.52 ± 2.134	46.31 ± 1.429	84.45 ± 0.7978	92.95 ± 0.9970**^#^	98.50 ± 0.2844

n = 6; group, **p < 0.05—CSEO treatment versus control group, ***p < 0.005—CSEO treatment versus reference group, *p < 0.005—CSEO treatment versus control group, **#*p* < 0.05—reference treatment versus control group, ***#*p* < 0.005—CSEO treatment versus control group, *#*p* < 0.05—CSEO treatment versus reference group. Tukey’s multiple comparison tests were used after the one-way ANOVA to analyse the data. Values of p < 0.05 are significant.

### 3.4 Body weight

The impact of CSEO therapy on body weight and feed intake was evaluated and contrasted with the reference and control groups. Table 3 illustrates that rats administered CSEO had a significant rise in body weight (229.3 ± 4.688 g; ***p < 0.005) and feed intake (12.94 ± 0.6572 g; ***p < 0.005) compared to the control group (202.2 ± 4.110 g and 9.263 ± 0.2971 g, respectively). Moreover, in comparison to the reference group (204.7 ± 1.856 g and 11.00 ± 0.4343 g), the CSEO group exhibited a statistically significant enhancement in both parameters (#*p* < 0.05 for body weight and *p < 0.05 for feed intake). The results indicate that CSEO therapy enhances the treated rats’ physiological conditions and nutritional absorption.

**TABLE 3:** Impact of CSEO on body weight and feed consumption compared to the reference and control groups.

Groups	Body weight (grams)	Feed intake (grams)
CSEO	229.3 ± 4.688***	12.94 ± 0.6572***
Reference	204.7 ± 1.855***^#^	11.00 ± 0.4343*
Control	202.2 ± 4.111	9.263 ± 0.2971

n = 6; Values are presented as Mean ± SEM; ***p < 0.005 — CSEO treatment against the control group, ***#*p* < 0.05 — CSEO versus Reference group, *p < 0.05 — CSEO versus Reference group Data were analysed via one-way ANOVA, succeeded by Tukey’s multiple comparison test. P-values below 0.05 are considered significant.

### 3.5 Effect of CSEO on inflammatory markers and wound healing dynamics

The inflammatory cytokines TNF-α and IL-1β play pivotal roles in the regulation of wound healing, and their elevated expression is commonly associated with delayed tissue repair. In the current study, significantly increased levels of TNF-α (650.0 ± 42.82 pg/mg, **^#^ *p* < 0.05) and IL-1β (983.3 ± 60.09 pg/mg, *^#^ *p* < 0.05) were observed in the control group, indicating persistent inflammation and impaired healing. Treatment with CSEO markedly reduced TNF-α to 310.0 ± 21.91 pg/mg and IL-1β to 625.0 ± 30.96 pg/mg (***p < 0.005 vs control for both markers), suggesting potent anti-inflammatory activity. In comparison, the reference group, the treated group showed moderate decreases in TNF-α (450.0 ± 42.82 pg/mg, *p < 0.05) and IL-1β (825.0 ± 30.96 pg/mg, *p < 0.05). These results, presented in Table 4, clearly demonstrate the efficacy of CSEO in attenuating inflammatory responses through the downregulation of TNF-α and IL-1β, thereby promoting a favourable environment for effective wound repair.

**TABLE 4:** Effect of CSEO on CD68, TNF-α, and IL-1β against reference and control group.

Groups	CD68 (ng/mL)	TNF-α (pg/mg)	IL-1β (pg/mg)
CSEO	12.00 ± 0.5774***	310.0 ± 21.91***	625.0 ± 30.96***
Reference	15.83 ± 0.7032**	450.0 ± 42.82*	825.0 ± 30.96*
Control	31.82 ± 1.013***^#^	650 ± 42.83**^#^	983.4 ± 60.10*^#^

n = 6; The values are expressed as Mean ± SEM; ***p < 0.005 for CSEO versus control, ***#*p* < 0.005 for Reference versus control group, **p < 0.05 for CSEO versus Reference group, **#*p* < 0.005 for Reference versus control group, *#*p* < 0.005 for Reference versus control group, *p < 0.05 for CSEO against Reference. Data were analysed using one-way ANOVA and Tukey’s multiple comparison test. P-values below 0.05 are considered significant.

### 3.6 Effect of CSEO on CD68 level in experimental rats

CD68, a macrophage marker, was substantially increased in the control group (31.83 ± 1.014 ng/mL), indicating heightened macrophage infiltration and chronic inflammation in the wound tissue. In contrast, CSEO treatment significantly reduced CD68 levels to 12.00 ± 0.5774 ng/mL (***p < 0.005 vs control), demonstrating its role in limiting excessive macrophage recruitment. The reference group exhibited moderate CD68 expression (15.83 ± 0.7032 ng/mL, **p < 0.05). The results are highlighted in Table 4, the immunomodulatory properties of CSEO in regulating macrophage activity during the wound repair process.

### 3.7 Impact on oxidative stress indicators and antioxidant composition

**FIGURE 4:**
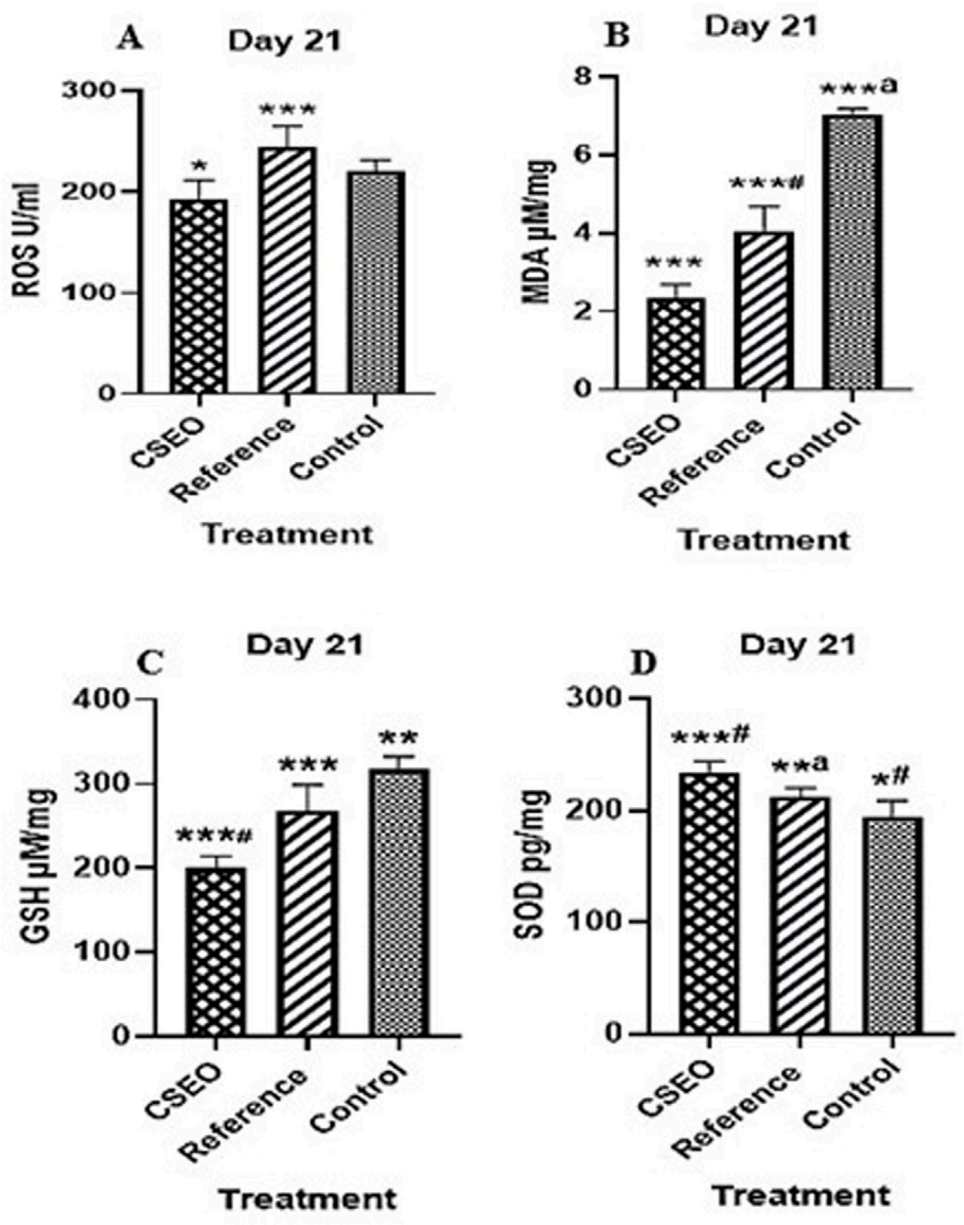
Impact of CSEO on **(A)** ROS, **(B)** MDA, **(C)** GSH, and **(D)** SOD in comparison to the reference and control groups. *n* = 6; Values are presented as Mean ± SEM; *p < 0.005—CSEO compared to control group, *#*p* < 0.05—Reference compared to control group, ***p < 0.05—CSEO compared to Reference group, ***#*p* < 0.005—CSEO compared to control group, ***^a^ *p* < 0.005—Reference compared to control group, **p < 0.05—Reference group compared to control, **^a^ *p* < 0.05—CSEO compared to Reference group. Data were analysed via one-way ANOVA, succeeded by Tukey’s multiple comparison test. P-values below 0.05 are deemed significant.

Figure 4 illustrates the protective effect of CSEO on oxidative stress markers, specifically (A) ROS, (B) MDA, (C) GSH, and (D) SOD, in comparison to both the reference and control groups. When compared to the control group, the CSEO-treated group showed a substantial decrease in ROS and MDA levels and a noteworthy rise in GSH and SOD levels (***p < 0.005). Moderate significance (*#*p* < 0.05) was found when comparing the reference and control groups.

Indicating partial protection by the reference treatment. Further comparisons demonstrated that CSEO was significantly more effective than the reference group in modulating oxidative stress parameters (*p < 0.05), with highly significant differences noted for certain markers (***^#^ *p* < 0.005). Moreover, substantial disparities between the reference and control groups (***^a^ *p* < 0.005 and **^a^ *p* < 0.05) were noted, affirming the validity of the experimental setup. These findings suggest that CSEO exerts a robust antioxidative effect, potentially superior to the reference, highlighting its therapeutic relevance in mitigating oxidative damage.

### 3.8 Histological results

#### 3.8.1 Measurement of histopathological changes

**FIGURE 5:**
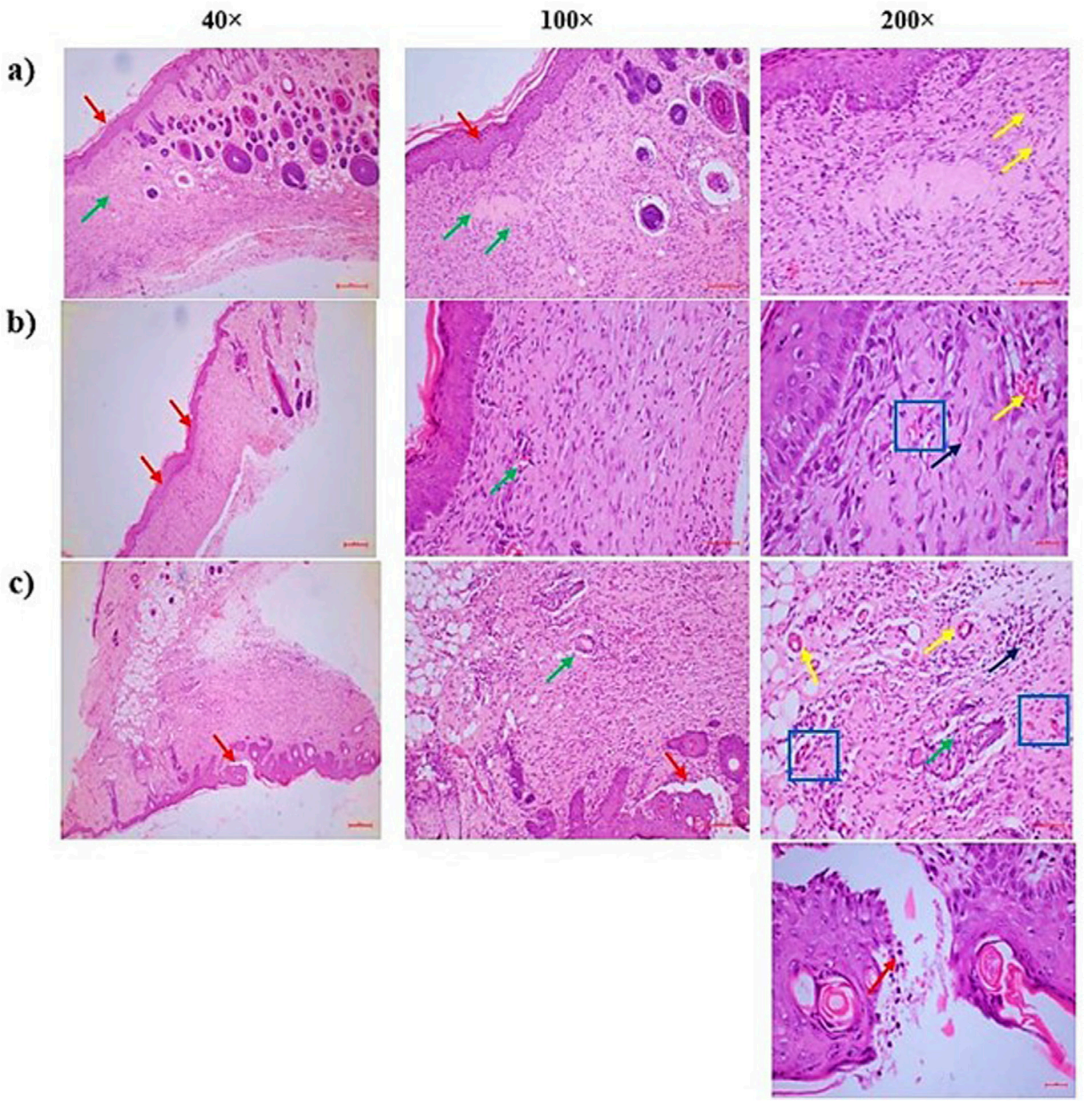
**(a)** CSEO treatment—There were three distinct findings: severe angiogenesis or neovascularisation [more than 15 blood vessels one HPF]- [Yellow arrow], partial re-epithelialization of the wound region with epidermal hyperplasia [Red arrow], and a moderate inflammatory response with granulation tissue proliferation in the dermal region surrounding the wound [Green arrow]. It was altered in the vicinity of the epidermis. Thick granulation tissue consists of a network of small blood vessels [yellow arrow: capillaries], fibroblasts [blue square] that produce collagen and other components of the extracellular matrix, and various inflammatory cells [black arrow], primarily plasma cells and lymphocytes, followed by neutrophils, **(b)** Reference—In the dermal region surrounding the wound, there was granulation tissue proliferation [Green arrow], mild neovascularisation or angiogenesis [less than five blood vessels one HPF]- [Yellow arrow], and the wound region was fully re-epithelialized with epidermal hyperplasia [Red arrow]. The wound region next to the epidermis was replaced. Thick granulation tissue is composed of a network of small blood vessels [yellow arrow: capillaries], fibroblasts [blue square] that produce collagen and other components of the extracellular matrix, and various inflammatory cells [black arrow], primarily plasma cells and lymphocytes, followed by neutrophils, **(c)** Control—The dermal region surrounding the wound showed a mild inflammatory response with granulation tissue proliferation [Green arrow], moderate angiogenesis or neovascularisation [5 to 10 blood vessels one HPF]- [Yellow arrow], and the wound region partially re-epithelialized with an excessive buildup of inflammatory exudates and necrotic materials in the epidermal layers [Red arrow]. The granulation tissue that replaced the wound area around the epidermal region is made up of a network of tiny blood vessels [capillaries, shown in yellow arrow], fibroblasts [blue square], which produce collagen and other extracellular matrix components, and different kinds of inflammatory cells [black arrow], primarily neutrophils, followed by plasma cells and lymphocytes.

Histopathological evaluation was conducted to assess tissue regeneration and repair in wound samples from all experimental groups. The analysis focused on five key parameters: collagen deposition, angiogenesis, granulation tissue development, re-epithelization, and inflammatory response. As shown in Table 5 and Figure 5, the CSEO-treated group exhibited marked improvements in tissue architecture and healing traits compared to the control and reference groups. Specifically, the inflammatory response and granulation tissue formation scored an average of 2 in the CSEO group, indicating moderate yet active tissue remodelling and immune cell infiltration. In contrast, the reference and control groups scored only 1, suggesting minimal or delayed inflammatory and granulation activity. Re-epithelization was also enhanced in the CSEO and reference groups with a score of 2, while the control group showed limited epithelial regeneration with a score of 1. Interestingly, angiogenesis, a critical factor in supplying nutrients and oxygen to healing tissue, was more prominent in the control group (score of 3), possibly due to compensatory mechanisms for poor healing, whereas the CSEO and reference groups showed regulated angiogenesis with scores of 1 and 2, respectively. Collagen deposition remained consistent across all groups, with a 1 score reflecting early or moderate matrix formation. These findings indicate that CSEO promotes an orchestrated and balanced wound healing process, particularly by enhancing granulation and re-epithelization while modulating angiogenesis and inflammation.

**TABLE 5:** CSEO caused histopathological alterations compared to the reference and control groups (*n* = 6).

Histopathology changes	Average score		
	CSEO	Reference	Control
Inflammatory Response	2	1	1
Granulation Tissue Formation	2	1	1
Re-epithelization	2	2	1
Angiogenesis	1	2	3
Collagen Deposition	1	1	1

## 4 Discussion

GC-MS analysis (Figures 1, 2; Table 1) of the CSEO revealed a distinctive chemical profile, notably rich in oxygenated monoterpenes and esters. The most abundant constituents were linalyl isobutyrate (18.88%), linalool (15.86%), alpha-terpineol (13.01%), and geranyl acetate (10.87%). Unlike conventional profiles often dominated by linalyl acetate, this sample lacked it entirely, instead displaying elevated levels of linalyl isobutyrate. This deviation suggests a phenotypic variation, likely influenced by geographical origin, harvest timing, climatic conditions, soil characteristics, or differences in distillation methods (Bhandari et al., 2024). Additional components, including caryophyllene, camphor, and beta-ocimene, contribute to the oil diverse and pharmacologically relevant composition. The unique phytochemical fingerprint of this sample may underlie its observed therapeutic efficacy.

Several major constituents identified in the CSEO, particularly linalool, alpha-terpineol, and geranyl acetate, are well-established for their powerful anti-inflammatory, antioxidant, and wound-repairing properties (Peana et al., 2002; Grazul et al., 2023). These pharmacological actions align closely with our experimental findings, including the downregulation of pro-inflammatory markers (IL-1β, TNF-α, and CD68), enhancement of antioxidant defences, a key transcription factor involved in redox homeostasis (Saha et al., 2020). Caryophyllene (6.72%), a sesquiterpene with known CB2 receptor-mediated anti-inflammatory effects, likely contributed to the immunomodulatory outcomes observed (Jha et al., 2021). Although direct evidence on linalyl isobutyrate remains limited, its close structural resemblance to linalyl acetate implies it may exert comparable biological effects through ester-linked mechanisms (Shenoy et al., 2011). This chemical synergy may account for the comprehensive therapeutic benefits seen in this study.

These findings highlight the diverse therapeutic potential of CSEO as a natural medication for enhancing wound healing. By improving diverse biochemical, histological, and physiological parameters, the oil demonstrates a holistic influence on the wound repair process validating its application in phytotherapeutic strategies targeting inflammatory and oxidative pathways (Shebaby et al., 2013; Ghaly et al., 2021). Derived from *Salvia sclarea*, CSEO has been historically valued for its anti-inflammatory, antimicrobial, and soothing properties. Recent research has highlighted its promising role in tissue repair and wound management (Ishfaq et al., 2018). The current study evaluated the effect of CSEO on key biological and physiological parameters associated with wound healing. These included body weight, feed intake, wound contraction rate, levels of inflammatory cytokines (IL-1β, TNF-α, CD68), antioxidant status, and histopathological changes. The results strongly support the hypothesis that CSEO enhances molecular and tissue-level healing processes.

Figure 3 represents images of wound healing across groups. One of the pivotal findings from this study was the notable improvement in body weight and feed intake among animals treated with CSEO (Table 3). These parameters are essential indicators of overall health during wound healing, as significant improvement in body weight reflects enhanced nutrient absorption and metabolic efficiency, which are vital for tissue repair. (Aćimović et al., 2018). CSEO bioactive components, such as linalool and linalyl acetate, are known for possessing anti-inflammatory and calming properties that reduce systemic stress, leading to better appetite and nutrient assimilation. (Kačániová et al., 2023). The observed improvement in body weight and feed intake, driven by the oil powerful modulation of inflammatory cytokines, further underscores its vital role in creating an ideal environment for effective healing. (Yücel and Özdemir, 2023). By enhancing nutritional status, CSEO supports the energy demands and cellular processes required for effective tissue regeneration, thus contributing to recovery. These findings align with prior research regarding the advantageous impacts of essential oils on wound healing and general health.

In terms of wound closure percentage, CSEO exhibited a remarkable ability to accelerate the rate at which wounds healed. Wound closure is a vital measure of effective healing, and the enhanced wound closure observed in this study (Table 2) highlights the potential of CSEO to stimulate cellular regeneration and repair. This action is probably due to its capacity to regulate essential metabolic processes involved in tissue repair, including the promotion of collagen synthesis and skin cell proliferation. (Guo et al., 2020). These findings suggest that CSEO helps close the wound more quickly and ensures that the healing process is progressing optimally, leading to improved wound outcomes. (Guo et al., 2020).

The anti-inflammatory properties of CSEO were notably demonstrated by the decrease in significant inflammatory markers, including TNF-α, IL-1β, and CD68, as seen in Table 4. These indicators are generally increased in reaction to tissue damage and are crucial in the inflammatory stage of wound healing. IL-1β and TNF-α are pro-inflammatory cytokines that, when present at high levels, can delay healing by promoting excessive inflammation. (Lieshchova et al., 2021). The downregulation of these markers in the CSEO group indicates its efficacy in modulating the inflammatory response, reducing chronicity, and facilitating a seamless transition of the wound healing process into the proliferative and remodelling stages. (Kohno et al., 2021).

CD68 marks monocytes and macrophages as two important immune cells that play a role in the inflammatory stage of wound healing. During wound healing, M1 macrophages (pro-inflammatory) dominate the early phase, producing cytokines like TNF-α and IL-1β to combat infection and clear debris. In the later stages of wound healing, M2 macrophages dominate, producing growth factors (e.g., VEGF, TGF-β) to support tissue repair and angiogenesis (Peana et al., 2002). Although VEGF and TGF-β were not evaluated in the current study, a notable reduction in IL-1β and TNF-α levels (Table 4) may suggest a diminished M1-driven inflammatory response. CD68 levels, measured in serum, were also reduced significantly in the CSEO-treated group (Table 4), indicating an overall attenuation of immune cell activity. However, it is important to acknowledge that macrophages do not exclusively express CD68; it is also found in other myeloid-lineage cells, including dendritic cells, fibroblasts, and neutrophils, which all contribute to inflammation (Khorsandi et al., 2022). Moreover, serum CD68 levels may not specifically reflect wound-site macrophage activity. Therefore, while the decrease in systemic CD68 suggests reduced overall inflammatory cell recruitment or activation, it cannot be definitively attributed to macrophage dynamics at the wound site. Future studies incorporating tissue-level assessments such as immunohistochemistry (e.g., DAB or fluorescence labelling) of wound biopsies would provide more precise insights into the cellular mechanisms underlying CSEO anti-inflammatory effects, including macrophage polarization and spatial distribution.

Linalool, a monoterpene alcohol present in CSEO, has significant antioxidant capabilities, rendering it essential for safeguarding cells against oxidative damage. Similarly, linalyl acetate, an ester, is known for its anti-inflammatory and antioxidant effects. (Yücel and Özdemir, 2023). Both compounds work synergistically to reduce the production of ROS at the wound site, thus preventing cellular damage that could otherwise delay healing. (Irshad et al., 2020). Additionally, these bioactive compounds contribute to the overall anti-inflammatory properties of CSEO, which is crucial in managing the early inflammatory phase of wound healing. (Gunaseelan et al., 2017).

As shown in Figure 4, the study observed reductions in ROS and MDA, along with increases in GSH and SOD, all of which are markers of improved antioxidant defense. Elevated levels of ROS and MDA are typically associated with oxidative stress, which can delay wound healing and cause tissue damage. In contrast, higher levels of GSH and SOD reflect a stronger defense against oxidative damage, suggesting that CSEO helps protect the wound area from oxidative injury and fosters a healthier environment for tissue regeneration (Sousa et al., 2020). These changes indicate a restoration of redox balance in the wound microenvironment, essential for preventing oxidative tissue damage and supporting tissue regeneration. Antioxidant responses are often regulated by key transcriptional factors, such as Nrf2, which modulates the expression of antioxidant enzymes. While the present study did not directly assess Nrf2 expression, the observed improvements in antioxidant markers like SOD and GSH suggest the involvement of Nrf2-mediated pathways in this process (Farkhondeh et al., 2020). This study highlights that CSEO provides a natural and effective approach to managing oxidative stress in wound healing.

Additionally, the study found significant improvements in histopathology, an essential process in wound healing where new epithelial cells cover the wound surface. The ability of CSEO to accelerate histopathological changes indicates that it plays a direct role in the regeneration of skin cells, which is crucial for the formation of a protective barrier over the wound. (Pastar et al., 2014). The study further supported this with a decrease in the histopathological changes, which signifies a reduction in wound severity and size over time, as demonstrated in Table 5 and Figures 5A–C. This effect is likely related to its regenerative properties, which help encourage the migration and growth of keratinocytes, the main cells that repair wounds. (Schultz et al., 2011).

Overall, the results of this study provide compelling evidence that CSEO offers a multifaceted approach to wound healing by attenuating oxidative stress and inflammation. Its ability to enhance wound closure, reduce inflammation, modulate oxidative stress, and promote re-epithelialization makes it an effective and natural adjunct to conventional wound care methods. By supporting the molecular and physiological aspects of healing, CSEO accelerates the recovery process, ensuring that wounds heal more quickly and effectively. However, while these results are promising, further clinical studies in human subjects are required to confirm the optimal dosing, application methods, and safety profiles of CSEO in wound healing. With continued research, CSEO may become a valuable tool in the management of wounds, providing a natural and effective solution for enhancing recovery.

Despite the encouraging outcomes, this study is not without limitations. The use of a rodent excision wound model, while well-established, may not fully represent the complex physiology and immune responses seen in human skin. Additionally, although systemic biomarkers such as serum IL-1β, TNF-α and CD68 were evaluated, local tissue-specific responses were not assessed using techniques such as immunohistochemistry or immunofluorescence. Such analyses would provide more precise insights into the wound-site cellular mechanisms, particularly macrophage polarization, fibroblast activation, and re-epithelialization dynamics. Furthermore, the current study did not explore important wound-healing mediators such as VEGF, TGF-β, Nrf2, and collagen synthesis. Future research should aim to address these aspects, including histological scoring, collagen quantification, and angiogenesis evaluation. Expanding this line of research through mechanistic studies and well-designed clinical trials will be essential to establish the translational potential, optimal formulation, and safety profile of CSEO in human wound care applications.

## 5 Conclusion

In conclusion, CSEO demonstrates promising potential in wound healing by reducing oxidative stress and exerting associated anti-inflammatory and tissue-regenerative effects. Research suggests that CSEO ability to promote circulation, reduce swelling, and regenerate skin cells can contribute to faster and more effective wound healing. While more clinical studies are needed to confirm its efficacy fully, CSEO offers a valuable, natural alternative or complement to traditional wound care methods.

## Data Availability

The raw data supporting the conclusions of this article will be made available by the authors, without undue reservation.
